# Targeting of phosphatidylserine by monoclonal antibodies enhances activity of immune checkpoint inhibitors in breast tumors

**DOI:** 10.1186/2051-1426-2-S3-P206

**Published:** 2014-11-06

**Authors:** Jian Gong, Van Nguyen, Shen Yin, Rich Archer, Emely Nguyen, Jeff Hutchins, Bruce Freimark

**Affiliations:** 1 Peregrine Pharmaceuticals, Inc, Tustin, CA, USA; 2 Department of Clinical Affairs, Peregrine Pharmaceuticals Inc., Tustin, CA, USA

## 

Phosphatidylserine (PS) is a phospholipid normally residing in the inner leaflet of the plasma membrane and becomes exposed on tumor vascular endothelial cells (ECs) and tumor cells. PS exposure becomes enhanced in response to chemotherapy, irradiation, and oxidative stresses in the tumor microenvironment. PS exposure in tumors promotes an immunosuppressive microenvironment which includes the recruitment of myeloid derived suppressor cells (MDSCs), immature dendritic cells, and M2-like macrophages as well as the production of anti-inflammatory cytokines [1]. In the present study, we evaluated the effect of a PS-targeting and anti-PD-1 antibodies in EMT-6 breast tumor model in Balb/c mice. Combination therapy inhibited tumor growth greater than the single agent therapies. Tumor growth inhibition correlates with infiltration of immune cells in tumors and induction of adaptive immunity. The combination of these mechanisms promotes strong, localized, anti-tumor responses without the side-effects of systemic immune activation. Our data demonstrate that combination of antibodies that block PS in combination with antibodies that block PD-1 may be promising therapy for breast cancer.
